# Icaritin Provides Neuroprotection in Parkinson’s Disease by Attenuating Neuroinflammation, Oxidative Stress, and Energy Deficiency

**DOI:** 10.3390/antiox10040529

**Published:** 2021-03-29

**Authors:** Hao Wu, Xi Liu, Ze-Yu Gao, Ming Lin, Xin Zhao, Yi Sun, Xiao-Ping Pu

**Affiliations:** 1 National Key Research Laboratory of Natural and Biomimetic Drugs, Peking University, Beijing 100191, China; 1310307406@bjmu.edu.cn (H.W.); 1611210107@bjmu.edu.cn (X.L.); zy_gao@bjmu.edu.cn (Z.-Y.G.); minglin@bjmu.edu.cn (M.L.); zhaoxin2010@bjmu.edu.cn (X.Z.); sunyi@bjmu.edu.cn (Y.S.); 2 Department of Molecular and Cellular Pharmacology, School of Pharmaceutical Sciences, Peking University, Beijing 100191, China

**Keywords:** icaritin, Parkinson’s disease, 1-methyl-4-phenyl-1,2,3,6-tetrahydropyridine, inflammation, oxidative stress, mitochondrial disfunction

## Abstract

Neuroinflammation, oxidative stress, and mitochondrial dysfunction are all important pathogenic mechanisms underlying motor dysfunction and dopaminergic neuronal damage observed in patients with Parkinson’s disease (PD). However, despite extensive efforts, targeting inflammation and oxidative stress using various approaches has not led to meaningful clinical outcomes, and mitochondrial enhancers have also failed to convincingly achieve disease-modifying effects. We tested our hypothesis that treatment approaches in PD should simultaneously reduce neuroinflammation, oxidative stress, and improve alterations in neuronal energy metabolism using the flavonoid icaritin in the 1-methyl-4-phenyl-1,2,3,6-tetrahydropyridine (MPTP) mouse model of PD. Using matrix-assisted laser desorption/ionization–mass spectrometry imaging (MALDI-MSI), coupled with biochemical analyses and behavioral tests, we demonstrate that icaritin improves PD by attenuating the the NOD-like receptor family pyrin domain-containing protein 3 (NLRP3) inflammasome activity and stabilizing mitochondrial function, based on our extensive analyses showing the inhibition of NLRP3 inflammasome, reduction of NLRP3-mediated IL-1β secretion, and improvements in the levels of antioxidant molecules. Our data also indicated that icaritin stabilized the levels of proteins related to mitochondrial function, such as voltage-dependent anion channel (VDAC) and ATP synthase subunit beta (ATP5B), as well as those of molecules related to energy metabolism, such as ATP and ADP, ultimately improving mitochondrial dysfunction. By employing molecular docking, we also discovered that icaritin can interact with NLRP3, VDAC, ATP5B, and several blood–brain barrier (BBB)-related proteins. These data provide insights into the promising therapeutic potential of icaritin in PD.

## 1. Introduction

The exact cause of neurodegenerative diseases, which are defined by common degenerative responses to diverse neurological processes, has not been fully elucidated [1]. Parkinson’s disease (PD) is a common neurodegenerative disease characterized by the preferential loss of dopaminergic neurons in substantia nigra and intracellular inclusions, and clinical symptoms include tremor, stiffness, and bradykinesia [2]. Substantia nigra is a part of midbrain, the top most structure present in the brain stem. It divides the cerebellar peduncles into anterior crus cerebri and posterior tegmentum of mid brain. The lesions in substantia nigra can cause a number of movement disorders including PD. PD affects 1% of individuals over the age of 60 years and 2–3% of those over the age of 65 years [3,4], highlighting the close relationship between PD and age. Importantly, the association between aging and increased inflammation has been well established. Aging is associated with increases in interleukin-1 beta (IL-1β), tumor necrosis factor-α (TNF-α), and activation of inflammasome complexes, such as the NOD-like receptor family pyrin domain-containing protein 3 (NLRP3) inflammasome [1,5]. In addition, global age-related systemic inflammation in the central nervous system (CNS) is a proposed common mechanism of degenerative disorders that negatively impacts the lifespan of elderly [6,7]. Elevated mitochondrial oxidant stress in human substantia nigra dopaminergic neurons triggers a dopamine-dependent toxic cascade, leading to lysosomal dysfunction and α-synuclein accumulation [8]. Moreover, evidence suggests that PD is associated with mitochondrial dysfunction and that the inhibition of complex I of the mitochondrial electron transport chain is selectively dysfunctional in dopaminergic neurons in sporadic PD [9]. Collectively, inflammation, oxidative stress, and mitochondrial dysfunction represent important regulators in PD. However, despite extensive efforts, various therapeutics that target inflammation, such as IL-1β-neutralizing antibodies and nonsteroidal anti-inflammatory drugs, have failed to induce the predicted clinical benefits [10]. Reduction of oxidative stress with antioxidants rasagiline have not convincingly or consistently demonstrated disease-modifying effects [11]. Recent trials using mitochondrial enhancers such as creatine and mitoquinone have also failed to convincingly demonstrate their disease-modifying effects [12]. The important roles of inflammation and mitochondrial abnormalities in neurodegeneration indicate that treatment approaches in PD should simultaneously reduce neuroinflammation and improve alterations in neuronal energy metabolism.

The classic experimental models of PD include toxic compounds aimed to reproduce the pathological and behavioral changes of the human disease in rodents, such as the pharmacological agent 1-methyl-4-phenyl-1,2,3,6-tetrahydropyridine (MPTP), which induces the selective degeneration of nigrostriatal neurons [2]. After crossing the blood–brain barrier, MPTP is converted to methyl-phenyl-pyridinium (MPP^+^) by monoamine oxidase (MAO)-B in glial cells; then, MPP^+^ enters mitochondria to inhibit the activity of respiratory chain complex I, thereby inducing the production of reactive oxygen species (ROS) and neuroinflammation, leading to the death of dopaminergic neurons [13]. The widely utilized regimen developed by Tatton and Kish involves daily injections of 30 mg/kg free base MPTP for five consecutive days in young adult C57BL mice; in this model, dopaminergic lesions stabilize by 21 days after the initiation of MPTP administration [14].

Icaritin is produced by the hydrolysis of icariin, which is a flavonoid extracted from *Epimedium sagittatum maxim*, and it has anti-inflammatory, antioxidant, and immune-improving effects [15,16]. Although icariin, which exhibits no biological activity in vitro, is generally considered to be the main active ingredient of *Epimedium* Linn., its pharmacological activity has been shown to be associated with its active metabolites [17]. Additionally, recent studies have shown that icariin can be metabolized into icaritin by human intestinal bacteria [18] and that it can improve mitochondrial damage in the CNS of mice in models of Alzheimer’s disease [19], suggesting that icaritin may delay the development and/or progression of neurodegenerative diseases by regulating energy metabolism. Nevertheless, no study to date has systematically evaluated the effects of icaritin and its influence on brain metabolism-related small molecules in PD mice. In the present study, we utilized matrix-assisted laser desorption/ionization (MALDI)–mass spectrometry imaging (MSI) in PD mice and demonstrated that icaritin significantly protected against neurodegeneration through the inhibition of oxidative stress, NLRP3 inflammasome activation, and the simultaneous improvement of mitochondrial dysfunction.

MALDI–MSI allows the simultaneous in situ detection of the levels and spatial distribution patterns of small molecules in cryosectioned tissues [20]. The technique is simple, rapid, and high-throughput with high accuracy. Using 1,5-diaminonaphthalene hydrochloride as a matrix, MALDI–MSI can detect various small molecules, including antioxidant molecules, energy metabolism-related molecules, amino acids, and metal ions in brain tissues [21], all of which are important for studying the roles of icaritin in PD.

## 2. Materials and Methods

### 2.1. Reagents and Drugs

**Figure 1:**
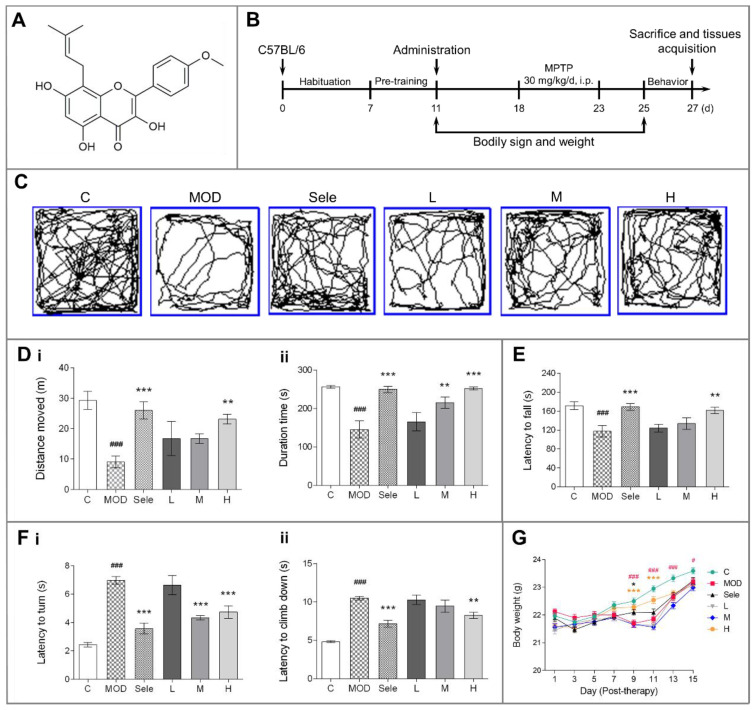
Effects of icaritin on motor dysfunction and body weight in mice in the 1-methyl-4-phenyl-1,2,3,6-tetrahydropyridine (MPTP) model of Parkinson’s disease (PD). (**A**) Chemical structure of icaritin. (**B**) Schedule of MPTP administration. The exploratory profile of experimental mice was determined with open field, rotarod, and vertical grid tests. (**C**,**D**) Trajectory map, distance moved (m), and duration (s) in the open field test. (**E**) Latency to fall (s) in the rotarod test. (**F**) Latency to turn (s) and climb down (s) in the vertical grid test. (**G**) Body weight. Data are presented as means ± SEM; n = 9–10. C: control; MOD: PD model; Sele: selegiline; L: 4.73 mg/kg icaritin; M: 9.45 mg/kg icaritin; H: 18.90 mg/kg icaritin. ^#^ *p* < 0.05, ^###^ *p* < 0.001 vs. C group; * *p* < 0.05, ** *p* < 0.01, *** *p* < 0.001 vs. MOD group.

1-Methyl-4-phenyl-1,2,3,6-tetrahydropyridine (MPTP) and 1,5-diaminonaphthalene were purchased from Sigma-Aldrich (St. Louis, MO, USA). Selegiline Hydrochloride Tablets (approval number: H20160342) were produced by Orion Corporation (Espoo, Finland). Nano icaritin aqueous solution (production batch No.: 11001) was produced by Lunan Pharmaceutical Group (Linyi, China) (Chemical structure of icaritin, Figure 1A). Rabbit antibodies against tyrosine hydroxylase (TH, ab112), NLRP3 (ab214185), and HIF-1α (ab2185) were obtained from Abcam, the succinate dehydrogenase subunit A (SDHA, 11998T) and voltage-dependent anion channel (VDAC, 4661T) rabbit antibodies were purchased from Cell Signaling Technology (Danvers, MA, USA), and mouse antibody against ATP synthase subunit beta (ATP5B, sc-55597) was obtained from Santa Cruz Biotechnology, Inc. (Santa Cruz, CA, USA). An ELISA kit for murine IL-1β was purchased from Abcam and a kit for murine TNF-α was purchased from ImmunoWay Biotechnology.

### 2.2. Animals Care

Sixty male age-matched C57BL/6 mice weighing 20–22 g were purchased from Beijing Vital River Laboratory Animal Technology Co., Ltd. [Beijing, China; license No.: SCXK (Beijing) 2016-0011]. The mice were labeled, weighed and caged; water and food was provided ad libitum. Mice were maintained at 22 °C–24 °C and 50–60% humidity under a 12-h light/dark cycle for 1 week to acclimatise them before drug administration. All animal experiments were approved by the Peking University Biomedical Ethics Committee (Beijing, China; approval No. LA2017282) and conducted by experimenters who hold the employment certificate of the Department of Laboratory Animal Science, Peking University Health Science Center, China. All efforts were made to minimize animal suffering.

### 2.3. MPTP Mouse Model of PD

Mice were randomly divided into control, PD model, selegiline (15 mg/kg), low-dose icaritin (4.73 mg/kg), medium-dose icaritin (9.45 mg/kg), and high-dose icaritin (18.90 mg/kg) groups. One week before the MPTP treatment, the mice were intragastrically administered indicated selegiline or icaritin doses or an equal volume of deionized water for 15 days. To establish the PD model, from the 18th to 22nd day, MPTP (30 mg/kg, dissolved in 0.9% normal saline) was injected intraperitoneally for five consecutive days. The intragastric administration of selegiline or icaritin was performed one hour after the MPTP injection, and the control and PD model groups were intraperitoneally administered an equal volume of normal saline [22]. One day after the last injection, behavioral assessments were performed using the open field test, rotarod test, and vertical grid test. On the 27th day, all mice were sacrificed for further study. The body weights of the mice were recorded daily after administration (Figure 1B).

### 2.4. Behavioral Assessments

#### 2.4.1. Open Field Test

A white opaque plastic box (32 cm × 32 cm × 32 cm) was used for the open field [23]. Before evaluation, all mice were pre-adapted to the box for 2 min. Next, the mouse was placed in the center of the open field and video-recorded for 5 min. The box was cleaned with 75% alcohol between trials. The distance moved (m) and duration (s) in 5 min were scored.

#### 2.4.2. Rotarod Test

Mouse motor coordination was assessed by a rotarod apparatus (IITC Life Science, Woodland Hills, CA, USA) [23]. Before administration, all mice were trained on the rotarod (12 r/min, in 180 s linearly) for 180 s. This training process was performed for a minimum of three times to train all mice to walk on the rotarod. After the MPTP treatment, the rotarod test was conducted at a uniform acceleration speed of 180 s with 35 r/min, and the latency to fall was recorded.

#### 2.4.3. Vertical Grid Test

The vertical grid apparatus was a vertical placed open box (55 × 8 × 10 cm^3^), and the back side of the box was a metal grid [24]. During the test, the mouse was placed facing upward inside the apparatus, 3 cm from the top. Next, the mouse was allowed to turn around and climb down toward the bottom. The test was repeated if the mouse failed to climb down within 60 s. Three tests per mouse were performed with an interval of 10 min. Before the formal test, all mice were trained three times a day for three consecutive days. The entire test was recorded, and latency to turn (s) and latency to climb down (s) was determined.

### 2.5. Measurement of Serum IL-1β and TNF-α Levels

After behavioral assessments and anesthetization with 1% pentobarbital sodium (5 mL/kg), 1 mL of blood drawn from the abdominal aorta of each mouse was placed in a centrifuge tube and centrifuged at 3000 rpm for 15 min to obtain serum. The IL-1β and TNF-α concentrations in serum were determined using IL-1β and TNF-α test kits.

### 2.6. Measurement of Serotonin, DA, and the Metabolites in Striatum

The levels of dopamine (DA), and the metabolites 3, 4-dihydroxyphenylacetic acid (DOPAC), homovanillic acid (HVA), serotonin (5-hydroxytryptamine, 5-HT), and 5-hydroxyindoleacetic acid (5-HIAA) in the striatum of mice were analyzed by high-performance liquid chromatography (HPLC) using an electrochemical detector (BAS LC-4B, BASi, West Lafayette, IN, USA), as previously described [25]. The mobile phase was 10% methanol in 90% sodium citrate buffer (85 mM citric acid, 100 mM anhydrous sodium acetate, and 0.2 mM Na_2_EDTA; pH 3.68). The flow rate was 1.0 mL/min at 25 °C in the reversed phase column. After centrifugation (4 °C, 20,000× *g*, 20 min), 70 μL striatal tissue homogenate supernatant was injected directly into the HPLC system. Data were calibrated with an external standard. The levels of dopamine and serotonin as well as their metabolites were calculated and expressed as μg/g wet tissue weight.

### 2.7. Western Blotting Analysis

Midbrain tissue samples of mice were lysed in RIPA lysis buffer (50 mM Tris, pH 7.4, 150 mM NaCl, 1% NP-40 [catalog # FNN0021, Thermo Fisher Scientific], 0.5% sodium deoxycholate [catalog #, D6750; Sigma], and 0.1% sodium dodecyl sulfate [catalog # 74255; Sigma]), as previously described [26]. Total extracted protein amounts were determined by the BCA^TM^ protein assay kit (catalog# 23235; Thermo Fisher), and 30-μg protein aliquots were separated using sodium dodecyl sulfate-polyacrylamide gel electrophoresis with 10% gels. After separation, the proteins were electrophoretically transferred onto polyvinylidene fluoride membranes (catalog #, IPVH00010; Merck Millipore, Birrica, MA, USA). The membranes were blocked with 5% skim milk in Tris-buffered saline (20 mM Tris-HCl, 150 mM NaCl, pH 7.4) with 0.1% Tween 20 (catalog # T104863; Aladdin reagent Co., Ltd., Shanghai, China), followed by incubation with the following primary antibodies overnight at 4 °C: anti-tyrosine hydroxylase (TH), anti-NLRP3, anti-succinate dehydrogenase subunit A (SDHA), anti-voltage-dependent anion channel (VDAC), anti-ATP synthase subunit beta (ATP5B), and anti-hypoxia-inducible factor-1α (HIF-1α). These antibodies are all diluted at 1:1000. The membranes were incubated with peroxidase-conjugated secondary antibodies and visualized using enhanced chemiluminescence (Elpis-Biotech, Inc., Daejeon, Korea). Band intensities was quantified using the ImageJ software (NIH).

### 2.8. Molecular Docking

Molecular docking analyses were conducted using MOE v2019.1 [27]. The 3D structure of icaritin was downloaded from the PubChem database. The 3D structure of three target proteins was downloaded from the RCSB Protein Data Bank. Prior to docking, the AMBER10: EHT forcefield and the implicit solvation model of the Reaction Field (R-field) were selected.

The MOE-Dock program was used for molecular docking simulations of small molecules with the targets. The docking workflow followed the “induced fit” protocol, in which the side chains of the receptor pocket were allowed to move according to ligand conformations, with a constraint on their positions. The weight used for tethering side chain atoms to their original positions was 10. For each ligand, all docked poses were first ranked by the London dG scoring, and a forcefield refinement was performed for top 20 poses, followed by rescoring using GBVI/WSA dG. Molecular graphics were generated via MOE.

### 2.9. Matrix-Assisted Laser Desorption/Ionisation–Mass Spectrometry Imaging (MALDI-MSI)

Three mice each from the C, MOD, and H groups, were euthanized by intraperitoneal injection of a 3-fold anesthetic dose of 1% pentobarbital sodium (5 mL/kg), and the brains were quickly excised, snap frozen in liquid nitrogen, and stored at −80 °C.

Complete and smooth transverse frozen brain slices (thickness, 10 μm) were prepared using a cryostat microtome (Scotsman Jencons, Nussloch, Germany) at −17 °C. The substantia nigra was identified based on the *Mouse Brain in Stereotaxic Coordinates* (San Diego, CA, USA). And the slice position was −3.28 mm away from the bregma and 0.52 mm away from interaural midpoint in mice. Then, the tissue slices were transferred onto indium tin oxide-coated glass slides (Bruker Daltonics, Bremen, Germany) and desiccated in a vacuum pump for 30 min before matrix spraying. Next, the tissue slices were sprayed with an ImagePrep tissue imaging matrix sprayer (Bruker Daltonics, Billerica, MA, USA). The matrix solution was prepared as follows: 39.5 mg of 1,5-diaminonaphthalene was added to a solution of 500 µL of 1 M hydrochloric acid and 4 mL of deionized water. The mixture was ultrasonically dissolved until the visible particles disappeared. Then, high-purity anhydrous ethanol (4.5 mL) was added. The matrix was sprayed according to the following settings: spraying for 2 s, incubation for 20 s, and drying for 75 s, with 10 spraying cycles. After the end of a spray, the slide was sprayed a second time in the opposite direction.

Slices were analyzed by MALDI–MSI using an Autoflex Speed™ MALDI TOF (TOF) system with a 2-kHz Smartbeam-II laser (Bruker Daltonics, Bremen, Germany) according to the protocols used in previous studies [21,28]. The results of MALDI–MSI were analyzed according to the study by Liu and colleagues [28].

### 2.10. Statistical Analysis

Data are expressed as mean ± SEM. Statistical analyses of MALDI–MSI were performed using SCiLS Lab based on the normalization of total ion chromatography data. All data were analyzed using GraphPad Prism 6.0 (GraphPad Prism, CA, USA). All results were compared using one-way analysis of variance (ANOVA). Comparison between the groups was performed using Fisher’s least significant difference (LSD) test. *p* < 0.05 was considered significant.

## 3. Results

### 3.1. Icaritin Improves Motor Dysfunction and Body Weight in PD Mice

Patients with PD have tremors, stiffness, and bradykinesia. Therefore, we evaluated whether mice exhibited behavioral signs of PD after MPTP injection and assessed the efficacy of icaritin in improving behavioral coordination and muscle strength. The daily intragastric administration of icaritin (Figure 1A) was initiated 7 days before the MPTP challenge and continued for 3 days after the initiation of MPTP challenge, as shown in Figure 1B. The mice were sacrificed on day five post-dopaminergic lesion (the 27th day). We found that the MPTP-treated mice had deficits in the open field (Figure 1C and (Di–ii)), rotarod (Figure 1E), and vertical grid (Figure 1(Fi–ii)) tests, all of which were reversed by treatment with icaritin or selegiline, which inhibits MAO-B. Furthermore, a decrease in body weight was observed after the intraperitoneal injection of MPTP due to its toxic effects. As shown in Figure 1G, the body weight of mice after five days of intraperitoneal MPTP injection in the PD model group was significantly lower than that of mice in the control group after 9, 11, 13, and 15 days of continuous MPTP administration. In contrast, high-dose icaritin treatment increased body weight in mice after 9 and 11 days of continuous MPTP administration. Moreover, compared with the PD model group, selegiline inhibited the weight loss on the 9th day of continuous MPTP administration. Collectively, these results indicated that icaritin significantly improved the MPTP-induced behavioral abnormalities in mice. Behavioral disorders are closely related to the degradation of dopaminergic neurons; therefore, we next assessed whether icaritin improved dopaminergic neuron damage.

### 3.2. Icaritin Reduced Dopaminergic Neuronal Damage by Inhibiting NLRP3 Inflammasome Activation

**Figure 2:**
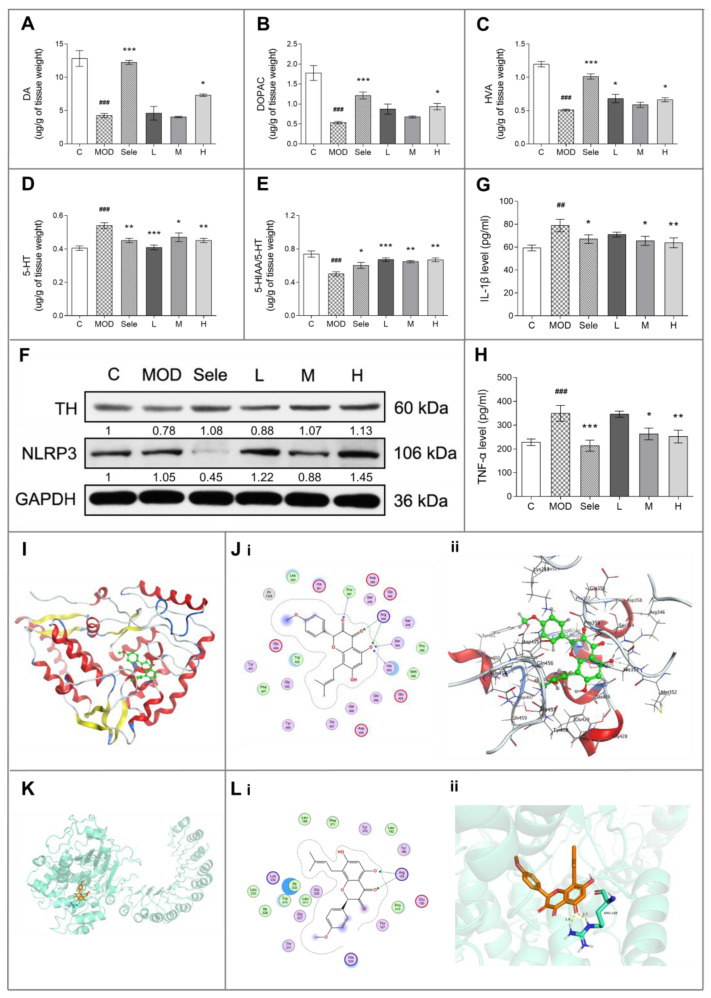
Icaritin inhibits NLR family pyrin domain containing 3 (NLRP3) activation and reduces dopaminergic neuron damage in mice in the 1-methyl-4-phenyl-1,2,3,6-tetrahydropyridine (MPTP) model of PD. (**A**–**E**) HPLC analysis to determine the levels of dopamine (DA), 3, 4-dihydroxyphenylacetic acid (DOPAC), homovanillic acid (HVA), and 5-hydroxytryptamine (5-HT), and to determine the 5-hydroxyindoleacetic acid (5-HIAA)/5-HT ratio. N = 3–4. (**F**) Western blot analysis of tyrosine hydroxylase (TH) and NLRP3 in the midbrain of mice treated with MPTP in the presence or absence of icaritin. Band intensity of the control group was set to 1, and relative values of other groups were calculated, presented under the blots. (**G**,**H**) Enzyme-linked immunosorbent analysis of serum interleukin-1 beta (IL-1β) and tumor necrosis factor-α (TNF-α) levels in the mouse model of MPTP-induced PD (*n* = 6). (**I**) Location of icaritin’s binding site on TH. (**J**) Docking result of icaritin with TH. (**i**) 2D, (**ii**) 3D; the green molecule is icaritin. (**K**) Location of icaritin’s binding site on NLRP3. (**L**) Docking result of icaritin with NLRP3. (**i**) 2D, (**ii**) 3D; the orange molecule is icaritin. Data in (**F**) are representative of three independent experiments with replications. Data are presented as means ± SEM. C: control; MOD: PD model; Sele: selegiline; L: 4.73 mg/kg icaritin; M: 9.45 mg/kg icaritin; H: 18.90 mg/kg icaritin. ^##^ *p* < 0.01, ^###^ *p* < 0.001 vs. C group; * *p* < 0.05, ** *p* < 0.01, *** *p* < 0.001 vs. MOD group.

Changes in the levels of dopamine and serotonin, major neurotransmitters related to neuronal function, suggest dopaminergic neuronal damage. MPTP injection promotes the degradation of dopamine and inhibits the activity of MAO-A to reduce serotonin reuptake, thereby reducing serotonin metabolism and increasing its levels in striatum [25]. DOPAC and HVA are dopamine metabolites, whereas 5-HIAA is a serotonin metabolite. Therefore, we used HPLC to determine the levels of dopamine and serotonin as well as their metabolites in the striatum of PD mice. Treatment with icaritin or selegiline reversed the reduction in the levels of DA, DOPAC, HVA, and 5-HIAA/5-HT (Figure 2A–C,E) and inhibited the increase in 5-HT levels (Figure 2D). Importantly, the selective inhibition of MAO-B by 15 mg/kg selegiline was too weak to result in serotonin syndrome [29]. Therefore, selegiline was more likely to restore serotonin levels by reducing neuronal damage. All together, these results indicated that icaritin improved the alterations in dopamine and serotonin metabolism in the mouse midbrain induced by MPTP injection. In addition, the TH protein expression levels in the midbrain extracts were upregulated by the icaritin treatment (Figure 2F and Supplementary Materials Figure S1), providing further evidence for the neuroprotective effect of icaritin.

We next conducted docking simulation studies to investigate the binding mode of icaritin with the TH protein (PDB ID: 2XSN) (Figure 2I–J). The oxygen atom of the carboxyl group in icaritin, which was considered as a hydrogen-bond acceptor, formed hydrogen bonds with Ser354 and His353 in TH. Additionally, the other oxygen atom of the carboxyl group in icaritin formed a hydrogen bond with Arg345 in TH. The docking score of icaritin, i.e., the S value, was −7.7489, and the binding free energy of the refined docking mode was −156.926 kcal/mol, which indicated that icaritin interacted very well with TH.

Conversely, inflammation has been increasingly demonstrated to contribute to the development of degenerative changes in the CNS, whereas subsequent increases in the levels of inflammatory cytokines, such as IL-1β and TNF-α, have been proposed to be pathological drivers of neurodegenerative diseases [1,5]. Therefore, we also investigated the effect of icaritin on neuroinflammation by measuring IL-1β and TNF-α serum levels in PD mice. As shown in Figure 2G,H, the icaritin treatment effectively inhibited the MPTP-induced increases in IL-1β and TNF-α serum levels, indicating that icaritin reduced neuroinflammation in this model of PD. Moreover, docking simulation studies indicated that icaritin might have a medium affinity with IL-1β and TNF-α (Figure S2, Table 1 and Tables S1–S2).

Activation of pattern recognition receptors is important in sterile inflammatory response to endogenous factors during neuroinflammation [30]. NLRP3 inflammasomes have been shown to function in the CNS, with the highest NLRP3 inflammasome expression observed in microglia [31]. Studies have shown that NLRP3 activation promotes IL-1β secretion [5]. Therefore, we elucidated whether icaritin protected against inflammation through the modulation of NLRP3 inflammasome activation in PD mice. As shown in Figure 2F and Figure S1, medium-dose icaritin reduced MPTP-induced NLRP3 protein expression, partially inhibiting NLRP3 activation. To investigate the binding mode of icaritin with the protein NLRP3 (PDB ID: 6NPY) (Figure 2K,L), docking simulation studies were carried out via MOE. The best docking score (S value) of the icaritin in NLRP3 is −8.118. The binding free energy of refined docking result is −134.135 kcal/mol (Table 1). This computational result indicates that icaritin may have a strong affinity with NLRP3.

These results indicated that icaritin inhibited NLRP3 activation and reduced IL-1β release, thereby attenuating neuroinflammation and dopaminergic neuronal damage to improve motor function in PD mice. Particularly, treatment with high-dose icaritin exhibited the most evident neuroprotective effects. Therefore, the high dose of 18.90 mg/kg was selected to further investigate the effect of icaritin on the levels of metabolism-related molecules in the substantia nigra of PD mice using MALDI-MSI.

### 3.3. Icaritin Increases the Level of Antioxidant Molecules and Regulates Glutamate–Glutamine Cycle in the Substantia Nigra, a motor nucleus present in the midbrain

**Figure 3:**
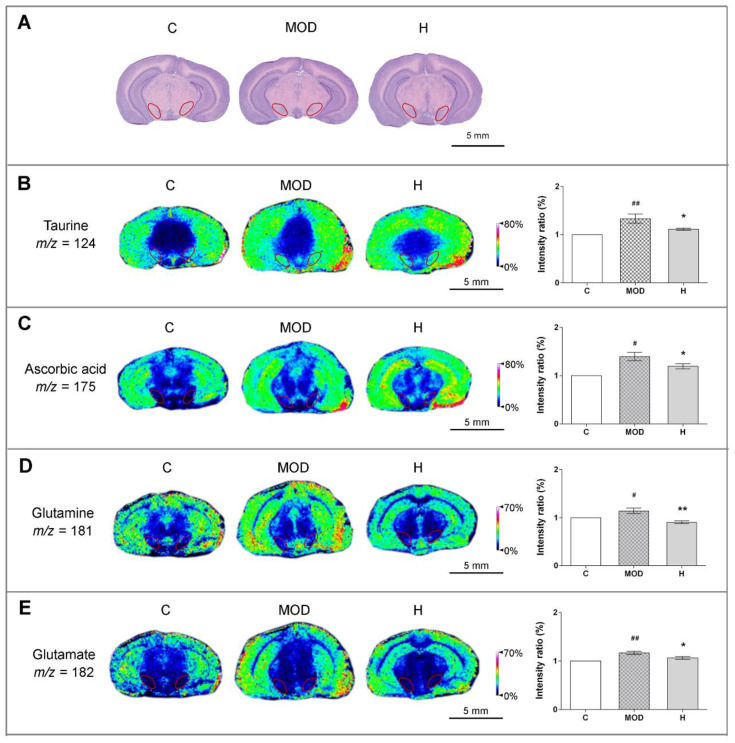
Icaritin attenuates the levels of antioxidant molecules in the substantia nigra of mice in the MPTP model of PD. (**A**) Hematoxylin/eosin staining of adjacent brain sections, and substantia nigra is marked by red circles, the position of which was −3.28 mm away from the bregma and 0.52 mm away from in-teraural midpoint in mice. (**B**–**E**) In situ matrix-assisted laser desorption/ionization–mass spectrometry imaging (MALDI–MSI) of taurine, ascorbic acid, glutamine, and glutamate. Spatial resolution: 200 μm; scale bar: 5 mm; *m*/*z*: mass-to-charge ratio. C: control; MOD: PD model; H: 18.9 mg/kg icaritin. Data are presented as means ± SEM; *n* = 3 per group. ^#^ *p* < 0.05, ^##^ *p* < 0.01 vs. S group; * *p* < 0.05, ** *p* < 0.01 vs. MOD group.

To further confirm the anti-inflammatory effects of icaritin in PD mice, we measured the levels of antioxidant molecules in substantia nigra using MALDI-MSI. As marked by red circles on MSI images in Figure 3A, the substantia nigra was identified based on the *Mouse Brain* *in Stereotaxic Coordinates* (San Diego, California, USA). The slice position was −3.28 mm away from the bregma and 0.52 mm away from interaural midpoint in mice, and adjacent brain sections were stained by hematoxylin/eosin. Studies indicate that taurine and ascorbic acid (vitamin C) might exert neuroprotection by removing ROS from the CNS [32,33]. As shown in Figure 3B,C, icaritin inhibited oxidative stress by attenuating the increases in the levels of taurine, ascorbic acid, and hypoxia-inducible factor-1α (HIF-1α) (Figure S3A) in the substantia nigra of PD mice. Additionally, our molecular docking analyses indicated that icaritin could interact with HIF-1α, with a low S value (−5.3245) and binding free energy (−74.195 kcal/mol) (Figure S3B,C).

In addition, glutamate and glutamine, excitatory amino acids present at high levels in the brain, participate in the glutamate–glutamine cycle and are important for maintaining normal brain function. Previous studies indicated that glutamate-mediated excitotoxicity was an important mechanism underlying the development of PD and that excess extracellular glutamate could be scavenged by antioxidant molecules [32,34]. As shown in Figure 3D–E, icaritin reversed the alterations in the levels of glutamate and glutamine in the substantia nigra of PD mice, further supporting that icaritin exerted neuroprotective effects.

Furthermore, considering that the tricarboxylic acid cycle can affect the metabolism of amino acids, alterations in the glutamate–glutamine cycle suggested abnormal energy metabolism; moreover, excessive ROS generation due to mitochondrial damage induced by MPTP injection was shown to be an important mediator of oxidative stress [12]. Thus, we next aimed to identify small molecules involved in the energy metabolism in substantia nigra to confirm the neuroprotective effect of icaritin.

### 3.4. Icaritin Ameliorates Mitochondrial Function in the Substantia Nigra

**Figure 4:**
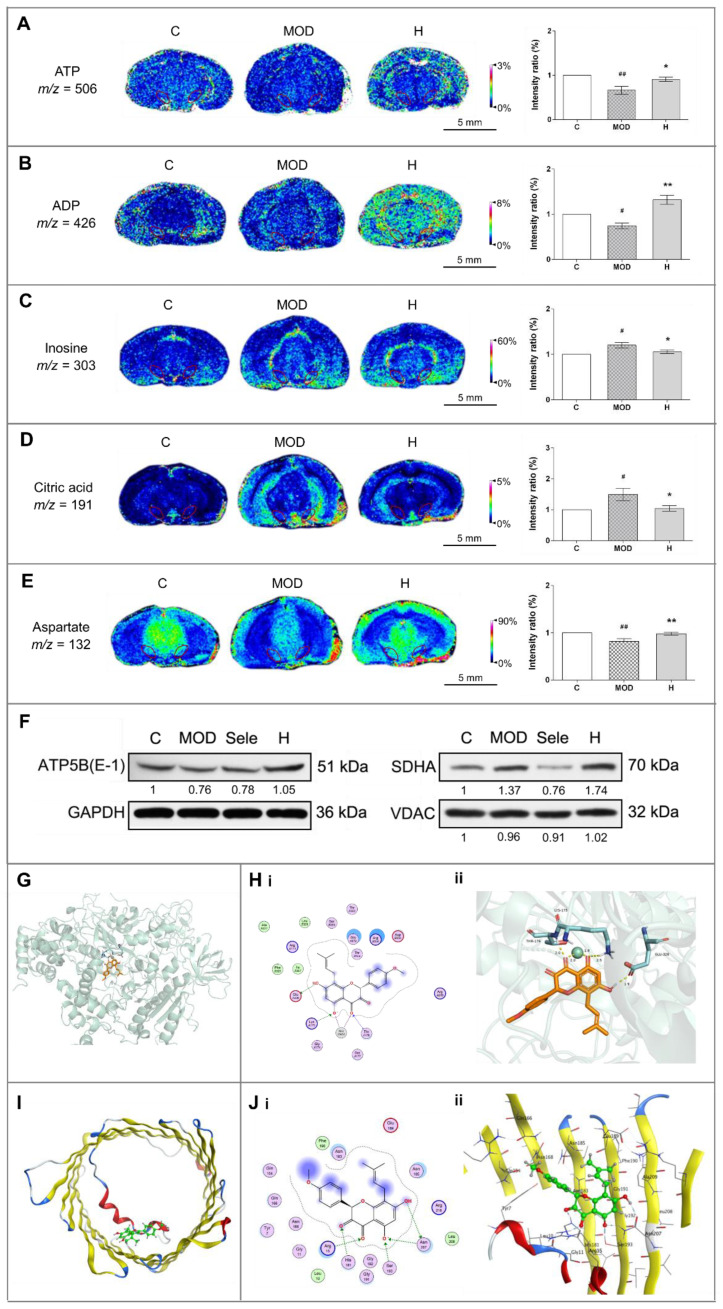
Icaritin augments ATP metabolism and stabilizes proteins related to mitochondrial function in the substantia nigra of mice in the MPTP model of PD. (**A**–**E**) In situ MALDI–MSI of ATP, ADP, inosine, citric acid, and aspartate. Spatial resolution: 200 μm; scale bar: 5 mm; *m*/*z*: mass-to-charge ratio. (**F**) Western blot analysis of ATP5B, SDHA, and VDAC in the midbrain of mice treated with MPTP in the presence of absence of icaritin. Band intensity of the control group was set to 1, and relative values of other groups were calculated, presented under the blots. (**G**) Docking result of icaritin in the ATP site of ATP5B. (**H**) Docking result of icaritin with ATP5B. (**i**) 2D, (**ii**) 3D; the orange molecule is icaritin. (**I**) Location of icaritin’s binding site on VDAC1. (**J**) Docking result of icaritin with VDAC1. (**i**) 2D, (**ii**) 3D; the green molecule is icaritin. Data in (**F**) are representative of three independent experiments with replications. C: control; MOD: PD model; H: 18.9 mg/kg icaritin. Data are presented as means ± SEM; *n* = 3 per group. ^#^ *p* < 0.05, ^##^ *p* < 0.01 vs. S group; * *p* < 0.05, ** *p* < 0.01 vs. MOD group.

Abnormal energy metabolism is an important driver t of PD [8]. Therefore, we examined whether icaritin could improve alterations in the levels of small molecules involved in energy metabolism in the substantia nigra. As shown in Figure 4A–D, icaritin reversed the changes in the levels of ATP, ADP, inosine, and citric acid in the substantia nigra of mice treated with MPTP, indicating that icaritin improved energy metabolism in PD.

As an important excitatory neurotransmitter, aspartate has a key role in the malate–aspartate shuttle, which can control the transport of nicotinamide adenine dinucleotide from cytoplasm to mitochondria for oxidative phosphorylation and ATP synthesis [35]. Thus, aspartate can be used as a potential indicator of energy metabolism; therefore, the increase in aspartate levels in mice administered MPTP and treated with icaritin demonstrated the recovery of mitochondrial energy metabolism (Figure 4E).

Energy metabolism is closely related to mitochondrial function. Thus, we next evaluated the changes in the levels of VDAC, SDHA, and ATP5B, which are related to mitochondrial function, to further investigate the effects of icaritin on mitochondrial functional stability in the substantia nigra of MPTP-administered mice. VDAC is an ion channel that facilitates the exchange of ions and molecules between mitochondria and cytosol [34], whereas SDHA and ATP5B are important components of the mitochondrial respiratory chain complexes. SDHA is involved in the electron transport chain, and ATP5B catalyzes ATP synthesis [35]. The reversal of VDAC and ATP5B levels by icaritin (Figure 4F and Figure S1) further suggested that icaritin could stabilize mitochondrial function to increase the energy supply.

There are two possible binding sites in the receptor in protein ATP5B: the binding site for ATP (Figure 4G,H) and the one for ADP (Figure S2). The computational result (Table 1) indicates that the icaritin mainly binds with ATP5B in ATP site; the interaction is quite strong. In mammalian cells, there are three VDAC isoforms: VDAC-1, which is the most widely expressed isoform, VDAC-2, and VDAC-3. The binding mode of icaritin with VDAC-1 is illustrated in Figure 4I,J. The oxygen atom in the benzene ring adjacent to pyrazine in icaritin formed two hydrogen bonds with Ser193 and Asn207 in VDAC-1. Icaritin could interact with VDAC-1 very well; the icaritin docking score (S value) was −5.8431, and the binding free energy of the refined docking result was −130.237 kcal/mol. Altogether, these results indicated that icaritin reduced damage of substantia nigra by stabilize mitochondrial function to improve energy metabolism.

### 3.5. Icaritin Might Interact with Blood–Brain Barrier Related Proteins to Improve PD Mice

Blood–brain barrier (BBB) homeostasis plays an important role in the treatment of PD, so we roughly verify the interaction between icaritin and BBB-related proteins, such as 12/15-lipoxygenase (12/15-LOX), occludin, claudin-5, and zonula occludens-1 (ZO-1). 12/15-LOX is a lipid-peroxidating enzyme that is triggered by ROS, and it can oxygenate free polyunsaturated fatty acids in biomembranes. The inhibition of 12/15-LOX was involved in the BBB protection [36]. Moreover, tight junction protein occludin, claudin-5, and ZO-1 are all tight junction proteins, which are involved in BBB function [37,38]. To investigate the binding mode of icaritin with these proteins (Figure S4), docking simulation studies were carried out via MOE. Via analyzing the best docking score and binding free energy (Table 1), we found that icaritin could interact with these proteins, indicating there is a certain relationship between icaritin and BBB function in PD protection.

## 4. Discussion

In the present study, we used an MPTP mouse model of PD to demonstrate that icaritin improved PD by attenuating NLRP3 inflammasome activity and stabilizing mitochondrial function. Our extensive analyses revealed that icaritin inhibited the NLRP3 inflammasome, reduced NLRP3-mediated IL-1β secretion, and regulated the levels of antioxidant molecules. We also demonstrated that icaritin stabilized the levels of proteins related to mitochondrial function, such as VDAC and ATP5B, as well as those of molecules related to energy metabolism, such as ATP, and ADP, ultimately improving mitochondrial dysfunction. Therefore, icaritin can exert an anti-PD effect by reducing dopaminergic neuronal damage and improving motor dysfunction in PD mice.

PD, the second most common neurodegenerative disease, is characterized by the preferential loss of dopaminergic neurons in the substantia nigra. Neuroinflammation has been demonstrated to sustain and exacerbate dopaminergic neuronal loss and dopamine deficiency [39], whereas NLRP3 inflammasomes are involved in the development of PD [40]. In the CNS, NLRP3 inflammasomes are activated by extracellular ATP, excess glucose, ceramides, and aggregated α-synuclein, among others [41,42]. IL-1β is cleaved from the inactive precursor pro-IL-1β by the NLRP3 inflammasome-activated caspase-1. Relatedly, high levels of IL-1β secreted in the CNS have been detected in patients with PD as well as in experiment animal models, indicating a close relationship between inflammation and neuronal damage in PD [10,42]. Thus, the NLRP3–IL-1β axis is a potential therapeutic target in PD. In the present study, we found that icaritin reversed the pathological increases in NLRP3 levels in the substantia nigra and IL-1β levels in serum. One study showed that the flavonoid kaempferol triggered the selective autophagic degradation of NLRP3 protein via ubiquitination, thus deactivating the NLRP3 inflammasome and inhibiting NLRP3-mediated IL-1β secretion [5]. Therefore, icaritin might also exert an anti-inflammatory effect through the attenuation of NLRP3 inflammasome activation.

Endogenous antioxidant molecules, such as taurine and ascorbic acid, can scavenge ROS and reduce oxidative stress. Studies have shown that taurine can be used for the treatment of PD by eliminating ROS in the CNS [32]. In addition, ascorbic acid has been shown to provide neuroprotection against glutamate-mediated excitotoxicity and ROS accumulation in PD [43]. An earlier study of Xu et al. indicated that icaritin protected mice from cerebral ischemic injury by inhibiting oxidative stress. Their further results indicate that icaritin can play a neuroprotective role against oxidative stress injury by activating Nrf2/Keap1 signaling pathway [44]. In the present study, we demonstrated that MPTP administration led to increases in glutamate and glutamine levels, which is consistent with a previous study [45], and promoted the enrichment of ascorbic acid in the substantia nigra. We also showed that icaritin reversed the alterations observed in the levels of HIF-1α, taurine, ascorbic acid, glutamic acid, and glutamine, which are related to reduction in ROS levels and stabilization of the glutamate–glutamine cycle; these results further confirmed that icaritin inhibited oxidative stress and neuroinflammation, reduced DA neuronal damage, and that it may delay the development of PD.

Increased ROS accumulation induced by mitochondrial dysfunction is an important cause of oxidative stress and inflammation in the CNS. In addition, mitochondrial dysfunction associated with partial deficiencies in respiratory chain complexes is implicated in age-related neurodegenerative diseases, including PD [46]. Respiratory chain complex I (nicotinamide adenine dinucleotide-ubiquinone oxidoreductase) deficiency is particularly implicated in PD [47]. Additionally, ATP deficiency induced by mitochondrial dysfunction leads to a decrease in vesicular dopamine uptake, resulting in increased dopaminergic degradation in neurons [48]. MPTP can inhibit complex I to suppress mitochondrial ATP production, leading to ROS overproduction and triggering a vicious circle between mitochondrial damage and oxidative stress [24,49]. This is the first study to demonstrate that icaritin treatment promotes the recovery of mitochondrial energy metabolism in the substantia nigra by reversing the changes in ATP and ADP levels. Subsequently, using VDAC as a marker, we found that the mitochondrial volume in the substantia nigra was reduced, based on the reduction in ATP5B levels. Therefore, as part of its anti-PD effects, icaritin can increase the levels of VDAC and ATP5B and mitochondrial volume, stabilizing mitochondrial function. In addition, we observed that the AMP/ATP ratio was increased significantly in the substantia nigra (Figure S5). An increase in the AMP/ATP ratio was previously reported to induce AMPK signaling pathway to exert neuroprotection [39,50]; therefore, the reversal of the increase in AMP/ATP ratio by icaritin suggests that icaritin might provide neuroprotection in PD by regulating the AMPK signaling pathway, which is a novel mechanism that requires further investigation.

Furthermore, there was moderate interaction between icaritin and several blood–brain barrier (BBB)-related proteins, such as 12/15- lipoxygenase, occludin, claudin-5, and ZO-1. We know that BBB homeostasis plays an important role in the treatment of PD, so there may be a positive correlation between icaritin and BBB function in PD protection, and this correlation deserves to be more fully demonstrated.

**Figure 5:**
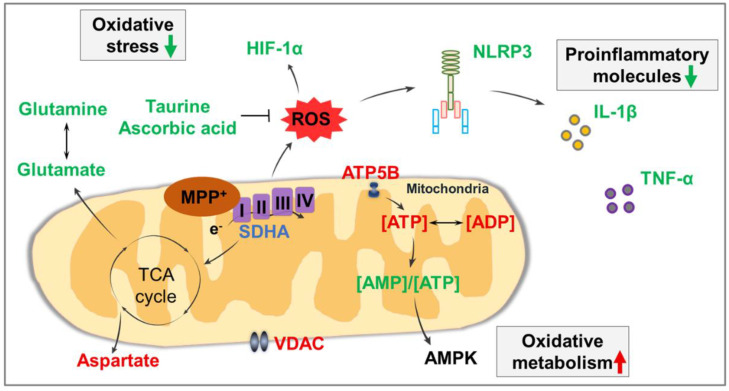
Summary of associations amongst affected molecules in the present study. Molecules that were significantly increased after icaritin treatment are represented in red font, those that were significantly decreased are represented in green font, and those that were significantly altered only in the presence of MPTP are represented in lilac font. Black arrows indicate metabolic processes. I–IV: respiratory chain complex I–IV; AMPK: AMP-activated protein kinase; HIF-1α: hypoxia-inducible factor-1α; ROS: reactive oxygen species; SDHA: succinate dehydrogenase subunit A; TCA cycle: tricarboxylic acid cycle; VDAC: voltage-dependent anion channel.

Altogether, the associations amongst these anomalous molecules are summarized in Figure 5. The administration of MPTP impairs the mitochondrial energy metabolism of dopaminergic neurons, leading to alterations in proteins related to mitochondrial function, small molecules related to energy metabolism, antioxidant molecules, and amino acids. Subsequently, increased ROS accumulation induces the activation of NLRP3 inflammasome, thereby promoting IL-1β secretion and dopaminergic neuronal damage, resulting in the development of PD. Thus, icaritin treatment can inhibit neuroinflammation, reduce oxidative stress, and improve mitochondrial function by modulating the levels of these molecules associated with the neuropathogenesis underlying PD.

## 5. Conclusions

In conclusion, our results in the present study provide strong evidence for the chemopreventive activity of icaritin against neurodegeneration in PD. The mechanisms underlying the activity of icaritin involve the stabilization of mitochondrial function by the regulation of mitochondrial function-related proteins, antioxidant molecules, energy metabolism-related small molecules, and amino acids, with the subsequent inhibition of NLRP3 inflammasome activation and reduction in IL-1β secretion, resulting in reduced neuroinflammation. The observed inhibition of neuroinflammation, reduction of oxidative stress, improvement of mitochondrial function, and interaction with BBB-related proteins, all of which are mediated by icaritin, illustrate the promising therapeutic potential of icaritin in PD.

## Figures and Tables

**Table 1:** The list of docking result.

ID	Molecules	Full Name of Target	Source	PDB ID ^1^	Docking Score (kcal/mol)	Binding Free Energy (kcal/mol)
1	icaritin	tyrosine hydroxylase	Homo	2XSN	−7.749	−156.926
2	icaritin	voltage-dependent anion channel 1	Mus	3EMN	−5.8431	−130.237
3	icaritin	hypoxia-inducible factor-1α	Mus	4ZPR	−5.3245	−74.195
4	icaritin	ATP synthase subunit 5 beta	Rat	1mab	−7.082(ADP site) −10.803(ATP site)	−49.804(ADP site) −344.802(ATP site)
5	icaritin	succinate dehydrogenase subunit A	Homo	6vax	−7.078	−159.603
6	icaritin	NLR family pyrin domain containing 3	Homo	6npy	−8.118	−134.135
7	icaritin	interleukin-1β	Mus	2mib	−6.217(site 1 in Table S1)	−65.829(site 1 in Table S1)
8	icaritin	tumor necrosis factor-α	Mus	2tnf	−7.440(site 1 in Table S2)	−52.203(site 1 in Table S2)
9	icaritin	12-lipoxygenase	Homo	3D3L	−4.83	−24.365
10	icaritin	15-lipoxygenase	Homo	1LOX	−8.67	−44.408
11	icaritin	occludin	Homo	1XAW	−6.94	−20.161
12	icaritin	claudin-5	Homo	6OV2	−6.10	−32.464
13	icaritin	zonula occludens-1	Homo	4OEO	−6.85	−32.049

^1^ PDB, Protein Data Bank.

## Data Availability

The data presented in this study are available within the article and in Materials.

## Supplementary Materials

Supplementary Materials can be found at https://www.mdpi.com/article/10.3390/antiox10040529/s1. Figure S1: Full gel scans for western blot, Figure S2: The binding mode of icaritin with protein IL-1β and TNF-α, Figure S3: Icaritin decreased the level of HIF-1α proteins in the substantia nigra of PD mice, Figure S4: The binding mode of icaritin with protein ALOX12, ALOX15, occludin, claudin-5, and ZO-1, Figure S5: Icaritin regulated AMP/ATP ratio in the substantia nigra of PD mice, Table S1: the top 5 binding site of icaritin to IL-1β, Table S2: the top 5 binding site of icaritin to TNF-α.

## References

[1] Goldberg E.L., Dixit V.D. (2015). Drivers of age-related inflammation and strategies for healthspan extension. Immunol. Rev..

[2] Blandini F., Armentero M.T. (2012). Animal models of Parkinson’s disease. FEBS J..

[3] Samii A., Nutt J.G., Ransom B.R. (2004). Parkinson’s disease. Lancet.

[4] Poewe W., Seppi K., Tanner C.M., Halliday G.M., Brundin P., Volkmann J., Schrag A.E., Lang A.E. (2017). Parkinson disease. Nat. Rev. Dis. Primers.

[5] Han X., Sun S., Sun Y., Song Q., Zhu J., Song N., Chen M., Sun T., Xia M., Ding J. (2019). Small molecule-driven NLRP3 inflammation inhibition via interplay between ubiquitination and autophagy: Implications for Parkinson disease. Autophagy.

[6] Youm Y.H., Grant R.W., McCabe L.R., Albarado D.C., Nguyen K.Y., Ravussin A., Pistell P., Newman S., Carter R., Laque A. (2013). Canonical Nlrp3 inflammasome links systemic low-grade inflammation to functional decline in aging. Cell Metab..

[7] Green D.R., Galluzzi L., Kroemer G. (2011). Mitochondria and the autophagy-inflammation-cell death axis in organismal aging. Science.

[8] Burbulla L.F., Song P., Mazzulli J.R., Zampese E., Wong Y.C., Jeon S., Santos D.P., Blanz J., Obermaier C.D., Strojny C. (2017). Dopamine oxidation mediates mitochondrial and lysosomal dysfunction in Parkinson’s disease. Science.

[9] Dawson T.M., Dawson V.L. (2003). Molecular pathways of neurodegeneration in Parkinson’s disease. Science.

[10] Rees K., Stowe R., Patel S., Ives N., Breen K., Clarke C.E., Ben-Shlomo Y. (2011). Non-steroidal anti-inflammatory drugs as disease-modifying agents for Parkinson’s disease: Evidence from observational studies. Cochrane Database Syst. Rev..

[11] Olanow C.W., Rascol O., Hauser R., Feigin P.D., Jankovic J., Lang A., Tolosa E. (2009). A double-blind, delayed-start trial of rasagiline in Parkinson’s disease. N. Engl. J. Med..

[12] Athauda D., Foltynie T. (2015). The ongoing pursuit of neuroprotective therapies in Parkinson disease. Nat. Rev. Neurol..

[13] Tatton N.A., Kish S.J. (1997). In situ detection of apoptotic nuclei in the substantia nigra compacta of 1-methyl-4-phenyl-1,2,3,6-tetrahydropyridine-treated mice using terminal deoxynucleotidyl transferase labelling and acridine orange staining. Neuroscience.

[14] Jackson-Lewis V., Przedborski S. (2007). Protocol for the MPTP mouse model of Parkinson’s disease. Nat. Protoc..

[15] Zhang W., Xing B., Yang L., Shi J., Zhou X. (2015). Icaritin Attenuates Myocardial Ischemia and Reperfusion Injury Via Anti-Inflammatory and Anti-Oxidative Stress Effects in Rats. Am. J. Chin. Med..

[16] Lai X., Ye Y., Sun C., Huang X., Tang X., Zeng X., Yin P., Zeng Y. (2013). Icaritin exhibits anti-inflammatory effects in the mouse peritoneal macrophages and peritonitis model. Int. Immunopharmacol..

[17] Kang H.K., Choi Y.-H., Kwon H., Lee S.-B., Kim D.-H., Sung C.K., Park Y.I., Dong M.-S. (2012). Estrogenic/antiestrogenic activities of a Epimedium koreanum extract and its major components: In vitro and in vivo studies. Food Chem. Toxicol..

[18] Wu H., Kim M., Han J. (2016). Icariin Metabolism by Human Intestinal Microflora. Molecules.

[19] Chen Y.J., Zheng H.Y., Huang X.X., Han S.X., Zhang D.S., Ni J.Z., He X.Y. (2016). Neuroprotective Effects of Icariin on Brain Metabolism, Mitochondrial Functions, and Cognition in Triple-Transgenic Alzheimer’s Disease Mice. CNS Neurosci. Ther..

[20] Smith A., L’Imperio V., Denti V., Mazza M., Ivanova M., Stella M., Piga I., Chinello C., Ajello E., Pieruzzi F. (2019). High Spatial Resolution MALDI-MS Imaging in the Study of Membranous Nephropathy. Proteom. Clin. Appl..

[21] Liu H., Li W., He Q., Xue J., Wang J., Xiong C., Pu X., Nie Z. (2017). Mass Spectrometry Imaging of Kidney Tissue Sections of Rat Subjected to Unilateral Ureteral Obstruction. Sci. Rep..

[22] Rai S.N., Birla H., Singh S.S., Zahra W., Patil R.R., Jadhav J.P., Gedda M.R., Singh S.P. (2017). Mucuna pruriens Protects against MPTP Intoxicated Neuroinflammation in Parkinson’s Disease through NF-kappaB/pAKT Signaling Pathways. Front. Aging Neurosci..

[23] Vitali R., Clarke S. (2014). Improved rotorod performance and hyperactivity in mice deficient in a protein repair methyltransferase. Behav. Brain. Res..

[24] Kim S.T., Son H.J., Choi J.H., Ji I.J., Hwang O. (2010). Vertical grid test and modified horizontal grid test are sensitive methods for evaluating motor dysfunctions in the MPTP mouse model of Parkinson’s disease. Brain. Res..

[25] Friedman L.K., Mytilineou C. (1990). Neurochemical and toxic effects of 1-methyl-4-phenyl-1,2,3,6-tetrahydropyridine and 1-methyl-4-phenylpyridine to rat serotonin neurons in dissociated cell cultures. J. Pharmacol. Exp. Ther..

[26] Zhang Y., Wang W., Zhang J. (2004). Effects of novel anxiolytic 4-butyl-alpha-agarofuran on levels of monoamine neurotransmitters in rats. Eur. J. Pharmacol..

[27] MOE (2019). 1010 Sherbooke St. West, Suite #910.

[28] Liu H., Chen R., Wang J., Chen S., Xiong C., Wang J., Hou J., He Q., Zhang N., Nie Z. (2014). 1,5-Diaminonaphthalene hydrochloride assisted laser desorption/ionization mass spectrometry imaging of small molecules in tissues following focal cerebral ischemia. Anal. Chem..

[29] Heinonen E.H., Myllyla V. (1998). Safety of selegiline (deprenyl) in the treatment of Parkinson’s disease. Drug. Saf..

[30] Patel M.N., Carroll R.G., Galván-Peña S., Mills E.L., Olden R., Triantafilou M., Wolf A.I., Bryant C.E., Triantafilou K., Masters S.L. (2017). Inflammasome Priming in Sterile Inflammatory Disease. Trends. Mol. Med..

[31] Kigerl K.A., de Rivero Vaccari J.P., Dietrich W.D., Popovich P.G., Keane R.W. (2014). Pattern recognition receptors and central nervous system repair. Exp. Neurol..

[32] Menzie J., Pan C., Prentice H., Wu J.Y. (2014). Taurine and central nervous system disorders. Amino Acids.

[33] Fernandez-Calle P., Jimenez-Jimenez F.J., Molina J.A., Cabrera-Valdivia F., Vazquez A., Garcia Urra D., Bermejo F., Cruz Matallana M., Codoceo R. (1993). Serum levels of ascorbic acid (vitamin C) in patients with Parkinson’s disease. J. Neurol. Sci..

[34] Kocot J., Luchowska-Kocot D., Kielczykowska M., Musik I., Kurzepa J. (2019). Does Vitamin C Influence Neurodegenerative Diseases and Psychiatric Disorders?. Nutrients.

[35] Yang H., Zhou L., Shimin Z., Zhao Y., Lin H., Zhang M., Zhao S., Yang Y., Ling Z., Guan K. (2015). SIRT3-dependent GOT2 acetylation status affects the malate-aspartate NADH shuttle activity and pancreatic tumor growth. EMBO J..

[36] Sun L., Yang L., Xu Y.W., Liang H., Han J., Zhao R.J., Cheng Y. (2012). Neuroprotection of hydroxysafflor yellow A in the transient focal ischemia: Inhibition of protein oxidation/nitration, 12/15-lipoxygenase and blood–brain barrier disruption. Brain. Res..

[37] Slava R., Nathan A.H., Sachin G., Seliga A., Reichenbach N.L., Persidsky Y. (2020). Hyperglycemia and advanced glycation end products disrupt BBB and promote occludin and claudin-5 protein secretion on extracellular microvesicles. Sci. Rep..

[38] Simona F.S., Sara M., Yasuteru S., Takashi K., Maria A.S. (2021). Protective effect of the sphingosine-1 phosphate receptor agonist siponimod on disrupted blood brain barrier function. Biochem. Pharmacol..

[39] Choi J.S., Park C., Jeong J.W. (2010). AMP-activated protein kinase is activated in Parkinson’s disease models mediated by 1-methyl-4-phenyl-1,2,3,6-tetrahydropyridine. Biochem. Biophys. Res. Commun..

[40] Yan Y., Jiang W., Liu L., Wang X., Ding C., Tian Z., Zhou R. (2015). Dopamine controls systemic inflammation through inhibition of NLRP3 inflammasome. Cell.

[41] Zhou Y., Lu M., Du R.H., Qiao C., Jiang C.Y., Zhang K.Z., Ding J.H., Hu G. (2016). MicroRNA-7 targets Nod-like receptor protein 3 inflammasome to modulate neuroinflammation in the pathogenesis of Parkinson’s disease. Mol. Neurodegener..

[42] Mamik M.K., Power C. (2017). Inflammasomes in neurological diseases: Emerging pathogenic and therapeutic concepts. Brain.

[43] Dawson T.M., Ko H.S., Dawson V.L. (2010). Genetic animal models of Parkinson’s disease. Neuron.

[44] Xu Y., Lu X., Zhang L., Wang L., Zhang G., Yao J., Sun C. (2021). Icaritin activates Nrf2/Keap1 signaling to protect neuronal cells from oxidative stress. Chem. Biol. Drug. Des..

[45] Bagga P., Pickup S., Crescenzi R., Martinez D., Borthakur A., D’Aquilla K., Singh A., Verma G., Detre J.A., Greenberg J. (2018). In vivo GluCEST MRI: Reproducibility, background contribution and source of glutamate changes in the MPTP model of Parkinson’s disease. Sci. Rep..

[46] Schapira A.H. (2016). Mitochondrial disease. Lancet.

[47] Muqit M.M., Gandhi S., Wood N.W. (2006). Mitochondria in Parkinson disease: Back in fashion with a little help from genetics. Arch. Neurol..

[48] Subramaniam S.R., Chesselet M.F. (2013). Mitochondrial dysfunction and oxidative stress in Parkinson’s disease. Prog. Neurobiol..

[49] Bueler H. (2009). Impaired mitochondrial dynamics and function in the pathogenesis of Parkinson’s disease. Exp. Neurol..

[50] Bayliss J.A., Lemus M.B., Stark R., Santos V.V., Thompson A., Rees D.J., Galic S., Elsworth J.D., Kemp B.E., Davies J.S. (2016). Ghrelin-AMPK Signaling Mediates the Neuroprotective Effects of Calorie Restriction in Parkinson’s Disease. J. Neurosci..

