# Supplementary material for: Icaritin Provides Neuroprotection in Parkinson’s Disease by Attenuating Neuroinflammation, Oxidative Stress, and Energy Deficiency
Source: Antioxidants (Basel). 2021 Mar 29;10(4):529. doi: 10.3390/antiox10040529 (PMC8066334; doi:10.3390/antiox10040529)
Supplement: Supplementary file 1 [file antioxidants-10-00529-s001.pdf]

antioxidants

MDPI

Supplementary Material

# Icaritin provides neuroprotection in acute Parkinson's disease by attenuating energy deficiency, oxidative stress, and neuroinflammation

**Hao Wu [1,2], Xi Liu [1,2], Ze-yu Gao [1,2], Ming Lin [1,2], Xin Zhao [1,2], Yi Sun [1,2] and Xiao-ping Pu [1,2,*]**

1 National Key Research Laboratory of Natural and Biomimetic Drugs, Peking University, Beijing 100191, China; 1310307406@bjmu.edu.cn (H.W.); 1611210107@bjmu.edu.cn (X.L.); zy_gao@bjmu.edu.cn (Z.-y.G.); minglin@bjmu.edu.cn (M.L.); zhaoxin2010@bjmu.edu.cn (X.Z.); sunyi@bjmu.edu.cn (Y.S.)
2 Department of Molecular and Cellular Pharmacology, School of Pharmaceutical Sciences, Peking University, Beijing 100191, China
* Correspondence: pxp123@bjmu.edu.cn; Tel.: +86-10-8280-2431

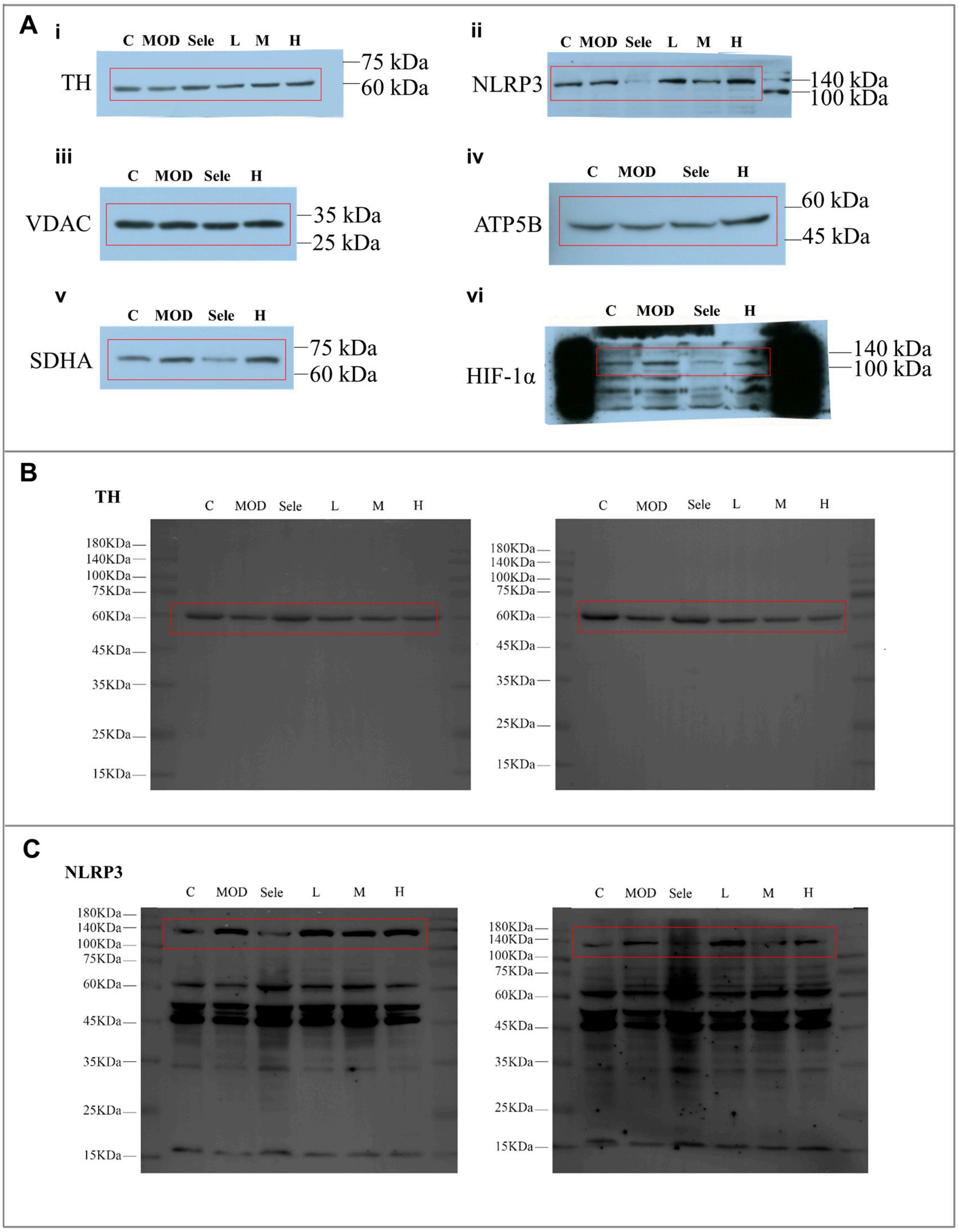
A
i
C MOD Sele L M H
TH
75 kDa
60 kDa
ii
C MOD Sele L M H
NLRP3
140 kDa
100 kDa
iii
C MOD Sele H
VDAC
35 kDa
25 kDa
iv
C MOD Sele H
ATP5B
60 kDa
45 kDa
v
C MOD Sele H
SDHA
75 kDa
60 kDa
vi
C MOD Sele H
HIF-1α
140 kDa
100 kDa
B
TH
C MOD Sele L M H
180KDa
140KDa
100KDa
75KDa
60KDa
45KDa
35KDa
25KDa
15KDa
C
NLRP3
C MOD Sele L M H
180KDa
140KDa
100KDa
75KDa
60KDa
45KDa
35KDa
25KDa
15KDa

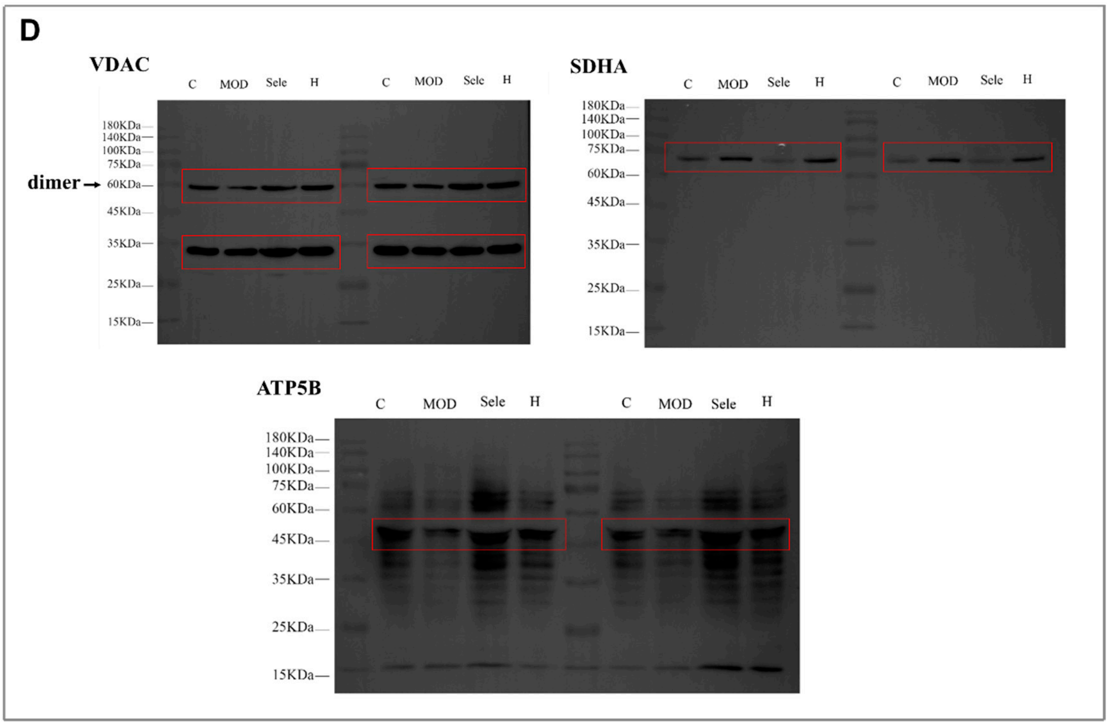


**Figure S1.** Full gel scans for western blot. A, gel scans for Fig. 2F(i-ii), 4F (iii-v), and Supplementary Figure 3A (vi). B, C, and D, the other two independent experiments with replications for TH, NLRP3, VDAC, SDHA, and ATP5B, respectively.

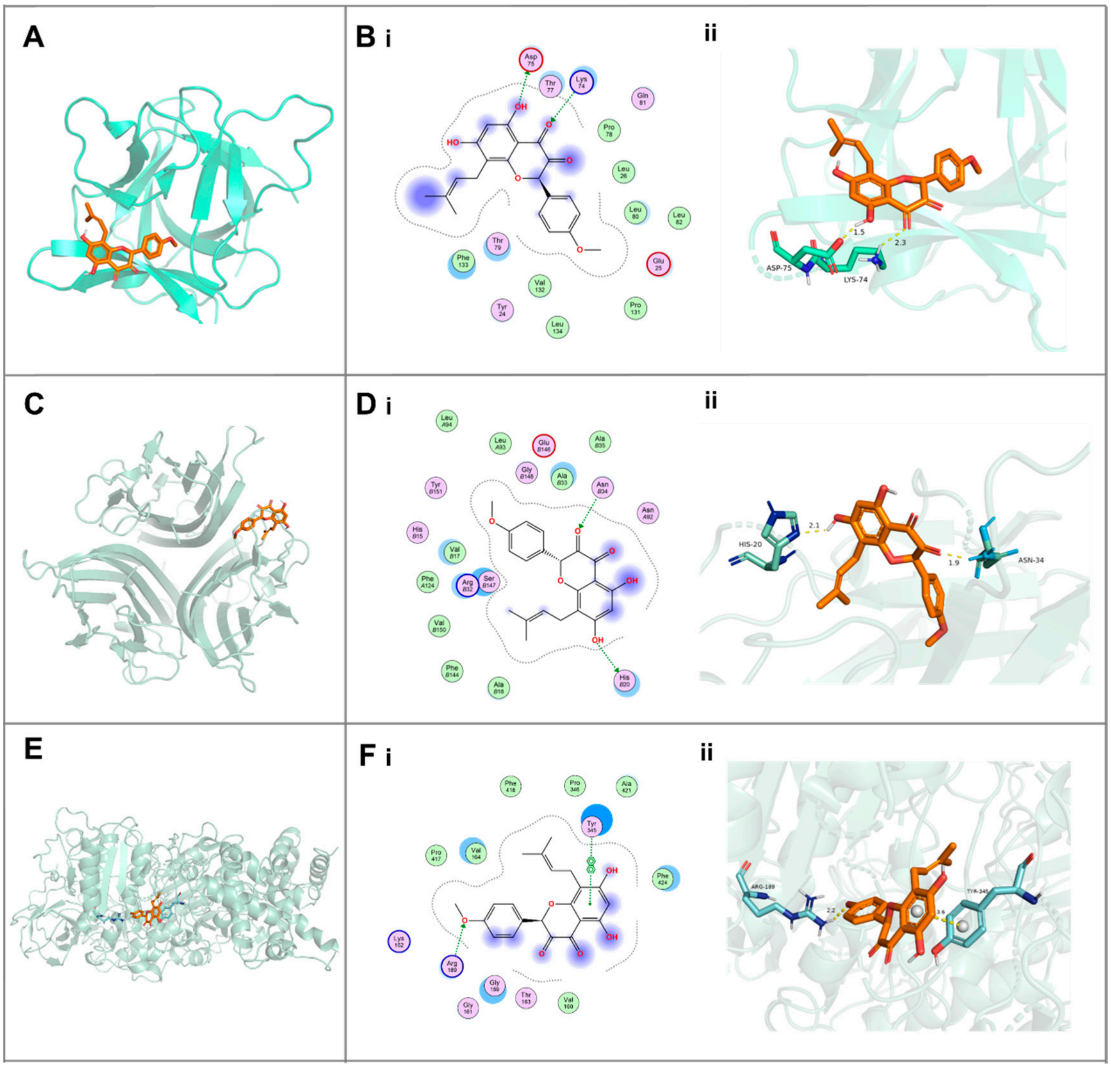


**Figure S2.** The binding mode of icaritin with protein IL-1β and TNF-α. (A), (C), and (E) Location of icaritin's binding site on IL-1β(site 1 in Supplementary Table 1), TNF-α. (site 1 in Supplementary Table 2), and ATP5B (the ADP site). (B), (D), and (F) Docking result of icaritin with IL-1β, TNF-α, and ATP5B. (i) is 2D, (ii) is 3D. The orange molecule is icaritin.

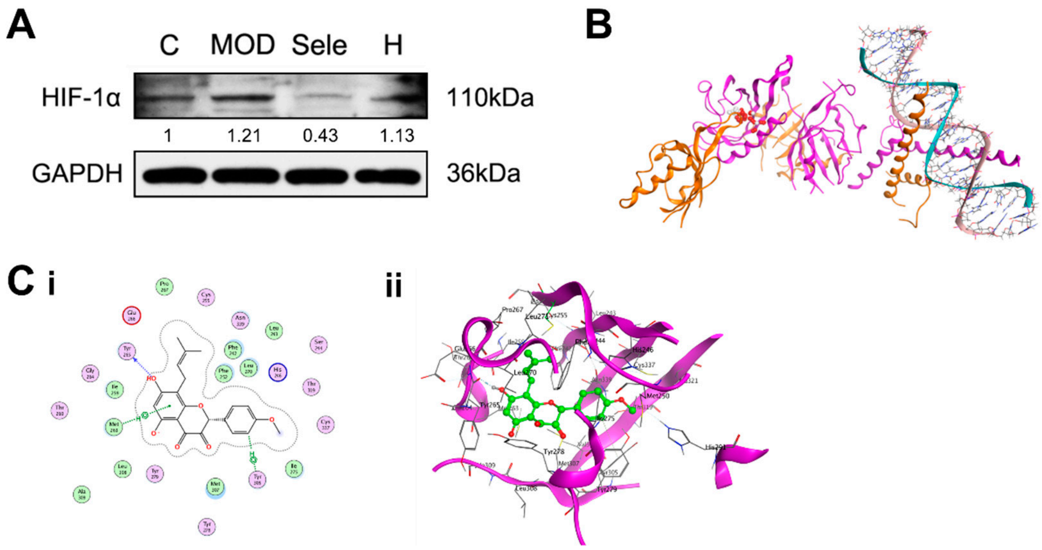


**Figure S3.** Icaritin decreased the level of HIF-1$\alpha$ proteins in the substantia nigra of PD mice. (A) Immunoblotting analysis of HIF-1$\alpha$ in the midbrain of mice treated with MPTP (or) plus icaritin. The gray value of the control group is set to 1, and the relative values of other groups are calculated. (B) Location of icaritin's binding site on HIF-$\alpha$. (C) Docking result of icaritin with HIF-$\alpha$. (i) is 2D, (ii) is 3D, and the green molecule is icaritin.

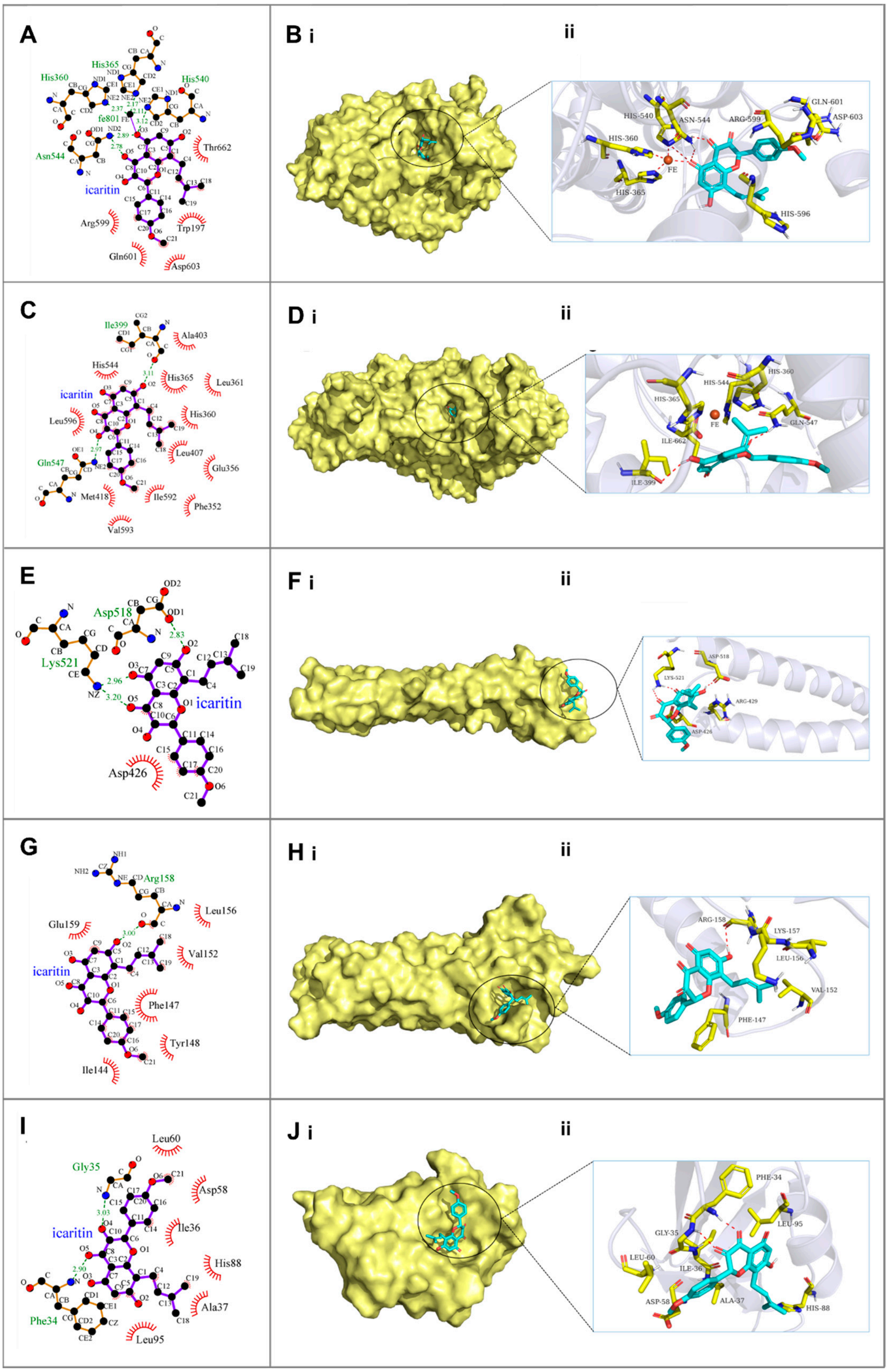

**Figure S4.** The binding mode of icaritin with protein ALOX12, ALOX15, occludin, claudin-5, and ZO-1. (A), (C), (E), (G), and (I) The 2D binding mode of icaritin with ALOX12, ALOX15, occludin, claudin-5, and ZO-1. (B), (D), (F), (H), and (J) Docking result of icaritin with ALOX12, ALOX15, occludin, claudin-5, and ZO-1. (i) is the binding mode, (ii) is 3D. Icaritin is colored in cyan, and the molecular surface of ALOX12, ALOX15, occludin, Claudin-5, and ZO-1 are colored in pale yellow.

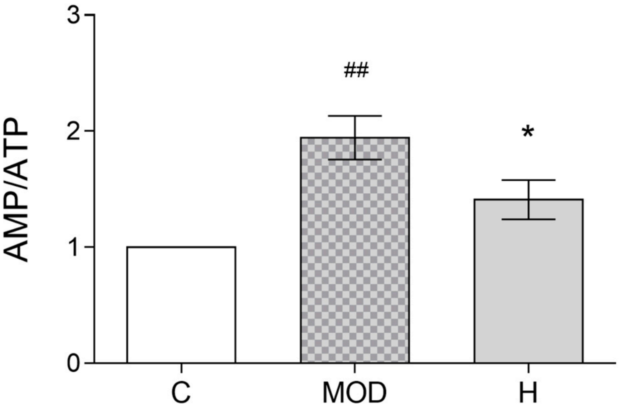


**Figure S5.** Icaritin regulated AMP/ATP ratio in the substantia nigra of PD mice. C, control group; MOD, PD model group; H, 18.9 mg/kg icaritin-treated group. Data are presented as mean ± SEM; n = 3 per group. ## *P*< 0.01 vs. S group; * *P* < 0.05 vs. MOD group.

**Table S1.** the top 5 binding site of icaritin to IL-1β

| Site | Size | Phospholamban | hydrogen | Side | Residues |
|---|---|---|---|---|---|
| 1 | 43 | 2.32 | 18 | 21 | 1:(TYR24 GLU25 LEU26 LEU69 PRO78 THR79 LEU80 GLN81 LEU82 PRO131 VAL132 PHE133 LEU134) |
| 2 | 29 | 0.18 | 7 | 16 | 1:(GLN5 HIS7 SER43 GLY61 LEU62 LYS63 GLY64 LYS65 ASN66 LEU67 TYR68 VAL85 ASP86 PRO87 TYR90 PRO91) |
| 3 | 35 | -0.03 | 18 | 19 | 1:(VAL19 LEU20 GLN38 GLN39 VAL40 ILE41 LEU62 LYS63 LYS65) |
| 4 | 11 | -0.38 | 9 | 14 | 1:(ARG4 GLN5 LEU6 MET44 SER45 PHE46 ILE56 VAL58 LYS103) |
| 5 | 9 | -0.61 | 5 | 10 | 1:(GLN5 LEU6 HIS7 TYR8 SER150 VAL151 SER152 NME153) |

**Table S2**. the top 5 binding site of icaritin to TNF-$\alpha$

| Site | Size | Phospholamban | hydrogen | Side | Residues |
|---|---|---|---|---|---|
| 1 | 315 | 4.09 | 62 | 114 | 1:(GLY68 CYS69 TYR72 LYS98 SER99 PRO100 CYS101 PRO102 LYS103 ASP104 THR105 PRO106 ALA109 GLU110 LEU111 LYS112 PRO113 TRP114 TYR115 GLU116)2:(GLY68 CYS69 TYR72 LYS98 SER99 PRO100 CYS101 PRO102 LYS103 ASP104 PRO106 GLU107 GLY108 ALA109 GLU110 LEU111 LYS112 PRO113 TRP114 TYR115 GLU116)3:(CYS69 TYR72 LYS98 SER99 PRO100 CYS101 PRO102 LYS103 ASP104 THR105 PRO106 GLU107 ALA109 GLU110 LEU111 LYS112 PRO113 TRP114 TYR115 GLU116) |
| 2 | 92 | 1.79 | 28 | 51 | 2:(LYS90 VAL91 ASN92 LEU93 PHE124)3:(HIS15 VAL17 ALA18 ASN19 HIS20 LEU29 ARG32 ALA33 ASN34 ALA35 ASP143 PHE144 ALA145 GLU146 SER147 GLY148 GLN149 VAL150 TYR151) |
| 3 | 70 | 1.04 | 24 | 47 | 1:(HIS15 VAL17 ALA18 ASN19 HIS20 ARG32 ALA33 ASN34 ALA35 ASP143 PHE144 ALA145 GLU146 SER147 GLY148 GLN149 VAL150)3:(LYS90 VAL91 ASN92 LEU93 PHE124) |
| 4 | 41 | 0.5 | 17 | 25 | 1:(GLU23 GLU24 LYS65 GLY66 GLN67 PRO139 LYS140 TYR141 LEU142 ASP143 PHE144 ALA145) |
| 5 | 56 | 0.41 | 22 | 38 | 1:(VAL91 ASN92 LEU93 PHE124)2:(HIS15 VAL17 ALA18 HIS20 ARG32 ALA33 ASN34 ALA35 ASP143 PHE144 GLU146 SER147 GLY148 GLN149 VAL150 TYR151) |
